# Wnt modulates MCL1 to control cell survival in triple negative breast cancer

**DOI:** 10.1186/1471-2407-14-124

**Published:** 2014-02-24

**Authors:** Lixin Yang, Aldwin Apollo Perez, Sayuri Fujie, Charles Warden, Jie Li, Yafan Wang, Bryan Yung, Yun-Ru Chen, Xiyong Liu, Hang Zhang, Shu Zheng, Zheng Liu, David Ann, Yun Yen

**Affiliations:** 1 Department of Molecular Pharmacology, Beckman Research Institute, City of Hope National Medical Center, Beckman Building, Room 4117, 1500 East Duarte Road, Duarte, CA 91010, USA; 2 Department of Molecular Medicine, Beckman Research Institute, City of Hope National Medical Center, 1500 East Duarte Road, Duarte, CA 91010, USA; 3 Translational Research Laboratory Core, Beckman Research Institute, City of Hope National Medical Center, 1500 East Duarte Road, Duarte, CA 91010, USA; 4 Cancer Institute (Key Laboratory of Cancer Prevention and Intervention, National Ministry of Education), Zhejiang University, Hangzhou, Zhejiang 310009, PR China

**Keywords:** WNT5B, MCL1, WNT/β-catenin pathway, Triple negative breast cancer (TNBC)

## Abstract

**Background:**

Triple negative breast cancer (TNBC) has higher rates of recurrence and distant metastasis, and poorer outcome as compared to non-TNBC. Aberrant activation of WNT signaling has been detected in TNBC, which might be important for triggering oncogenic conversion of breast epithelial cell. Therefore, we directed our focus on identifying the WNT ligand and its underlying mechanism in TNBC cells.

**Methods:**

We performed large-scale analysis of public microarray data to screen the WNT ligands and the clinical significance of the responsible ligand in TNBC. WNT5B was identified and its overexpression in TNBC was confirmed by immunohistochemistry staining, Western blot and ELISA. ShRNA was used to knockdown WNT5B expression (shWNT5B). Cellular functional alteration with shWNT5B treatment was determined by using wound healing assay, mammosphere assay; while cell cycle and apoptosis were examined by flowcytometry. Mitochondrial morphology was photographed by electron microscope. Biological change of mitochondria was detected by RT-PCR and oxygen consumption assay. Activation of WNT pathway and its downstream targets were evaluated by liciferase assay, immunohistochemistry staining and immunoblot analysis. Statistical methods used in the experiments besides microarray analysis was two-tailed t-test.

**Results:**

WNT5B was elevated both in the tumor and the patients’ serum. Suppression of WNT5B remarkably impaired cell growth, migration and mammosphere formation. Additionally, G0/G1 cell cycle arrest and caspase-independent apoptosis was observed. Study of the possible mechanism indicated that these effects occurred through suppression of mitochondrial biogenesis, as evidenced by reduced mitochondrial DNA (MtDNA) and compromised oxidative phosphorylation (OXPHOS). *In Vivo* and *in vitro* data uncovered that WNT5B modulated mitochondrial physiology was mediated by MCL1, which was regulated by WNT/β-catenin responsive gene, Myc. Clinic data analysis revealed that both WNT5B and MCL1 are associated with enhanced metastasis and decreased disease-free survival.

**Conclusions:**

All our findings suggested that WNT5B/MCL1 cascade is critical for TNBC and understanding its regulatory apparatus provided valuable insight into the pathogenesis of the tumor development and the guidance for targeting therapeutics.

## Background

Triple negative breast cancer (TNBC) is an aggressive form of breast cancer characterized by the lack of estrogen, progesterone receptors (ER, PR) and lack of amplification of human epidermal growth factor receptor 2 (HER2) [1]. With the major contribution of adjuvant targeting therapies, the outcome of breast cancer has been improved dramatically; yet the prognosis of TBNC remains quite poor among the breast cancer subtypes [2-4]. It is largely due to the heterogeneous nature of TNBC and unresponsiveness to the clinic available targeting therapies [5-7]. Many attempts to identify the key oncogenic pathways at the molecular level have been carried out. Aberration of WNT signal is widely recognized as one of the potential pathway that contributes to TNBC tumorigenicity [8-11].

WNT and their downstream responsive genes modulate various processes that are crucial for development and growth, cell fate decision, cell proliferation/differentiation and stem cell self-renewal [12-14]. Activation of WNT signaling cascade is initiated through the binding of WNT with its receptor/co-receptor. WNT/β-catenin is the first indentified WNT pathway that is aberrantly activated in human colorectal cancer [15,16]. Since then, the complicated signals triggered by WNT, but following distinct pathways have been detected. The complexity of these signals is partially attributed to the multiple members of WNT family and various subtypes of receptor/co-receptor [17]. The cellular response to a given WNT ligand is ultimately context-specific and the dynamic interactions determine the net outcome [18]. Emerging evidence has been demonstrated that WNT signaling is actively involving in many cellular biologic processes via integrating WNT signal to other major cellular pathways, including mitochondrial homeostatic pathway [19].

Mitochondria engage in various biochemical activities and are the major organelle to generate ATP. In addition to their function as the power plants, they are involving in many other vital cellular processes, such as cell apoptosis, cell cycle control, cell differentiation and cell proliferation [20,21]. The functional and active mitochondria status is actually essential for cancer cell physiology. Despite frequent mitochondrial gene mutations are detected in human tumor; they don’t turn off the mitochondrial energy metabolism at all. Additionally, they regulate the mitochondrial bioenergetic and biogenetic state [22]. However, how cancer cells modulate mitochondrial status to meet their biological need is under current study. In the current project, we present evidence to demonstrate that MCL1 is a key regulator for TNBC cell survival mediated by controlling mitochondrial biogenesis.

## Methods

### Patients, tissues and serum

All tumor tissues and serum were collected under the Institutional Review Board (IRB)-approved protocols at City of Hope National Medical Center (COH) or Zhejiang University respectively. The patients were given informed consent. One hundred and forty-two breast tumor tissues, including 21 TNBC and 121 Non-TNBC tissues were collected for immunohistochemistry staining. We also collected 30 sera from TNBC and Non-TNBC patients respectively with the assistance from the COH Translational Research Laboratory Core for ELISA assay. Immunohistochemical staining and FISH confirmed that ER/PR/HER2 were negatively expressed, as assessed by pathologists in the Department of Pathology of COH.

### Microarray analysis

For differential expression analysis, differential expression *P*-values were determined via t-test in R [23]. Significant results are expected to show *P*-value < 0.05. Differential expression between TNBC and non-TNBC was determined using data from 3 cohorts (Chin et al. [24], expO (GSE2109), and TCGA [25]. Differential expression between patients that did or did not develop metastatic tumors was determined using 2 cohorts (expO (GSE2109), TCGA [25] for WNT5B and 1 cohort (Desmedt et al.) for MCL1. For survival analysis, differences in survival between “high” and “low” expression groups were visualized in Kaplan-Meier plots and compared using Cox regression analysis, with *P*-values calculated via log-rank test, using the ‘survival’ package in R (A Language and Environment for Statistical Computing). The disease-free survival of WNT5B was quantified independently for 2 cohorts (Desmedt et al. and Wang et al. [26,27]) respectively. And then meta-analysis was conducted by utilizing the same WNT5B probe (so that the signals would be comparable) for an 80 month observation period. The disease-free survival of MCL1 was analyzed by the same method using the cohort of Desmedt et al.

### RT-PCR, RT-qPCR and qPCR

Total RNA extraction from MDA-MB-231 was carried out using the RNeasy Mini Kit (Qiagen). For cDNA synthesis, total RNA (1 μg) was transcribed using random hexamers (Invitrogen, Carlsbad, CA), and SuperScript III reverse transcriptase (Invitrogen) following the manufacturer’s protocol. For quantification of OXPHOS-related genes, the cDNA amplication program included a denature at 95°C for 3 min, followed by 40 cycles of 95°C for 10 s; 58°C for 30 s. For MtDNA detection, total cellular DNA was isolated with DNAeasy Blood and Tissue Kit (Qiagen). Mitochondrial DNA content was determined by qPCR by using comparing the mitochondrially encoded Cox2 gene to an intron of the nuclear-encoded β-globin (HBB) gene. All qPCR was performed using an iQ5 iCycler (Bio-Rad) according to the manufacturer’s instructions. Data were analyzed using Bio-Rad iQ5 Optical System Software v2.0. All products yielded a single band with the predicted size. All primers are listed in Additional file 1: Table S1 and all products yielded a single band with the predicted size.

### Western blot analysis

Cell protein was extracted from cells using RIPA buffer (Cell Signaling, Danvers, MA) with phosphatase inhibitor (Roche, Indianapolis, IN). Equal amount of protein was loaded and separated by SDS-PAGE. After the protein was transferred onto a membrane, the blot was blocked with 5% non-fat milk in TBS and probed overnight at 4°C using the following antibodies: WNT5B (Sigma-Aldrich, St. Louis, MO), Cyclin E (M20), TOM20 (F-10), Myc (9E10), AIF (E-1), MCL1 (S-19) (Santa Cruz Biotechnology, Santa Cruz, CA), Caspsae-3, Caspase-8,PGC-α, Cyclin D1 (Cell Signaling, Danvers, MA) and β-actin (Bio-Rad, Hercules, CA). Appropriate antibodies were used for secondary antibody reaction. Signal was detected by the ECL Plus Western Blot Detecting System (GE Healthcare, Piscataway, NJ).

### Cell culture and growth assays

The triple negative cell lines MDA-MB-231was purchased from ATCC and cultured in the recommended media. Specific lentivirus shRNA (Sigma-Aldrich) was used to disrupt the expression of WNT5B while shRNA targeting non-mammalian sequence (Sigma-Aldrich) served as control. WNT5B expression was determined by immunoblot analysis. MDA-MB-231 cells that expressed WNT5B or control shRNA (shWNT5B or shCtl cells) were cultured in growth medium to observe cell growth. Cells (3 × 10^4^/ml) were seeded into 24-well plates, and cell number was counted every day for five days using a Cellometer Auto T4 (Nexcelom Bioscience, Lawrence, MA). Independent experiments were performed in triplicate.

### Cell morphology, invasion

Cells were infected with shCtl or shWNT5B lentivirus and the morphology was observed and photographed after WNT5B expression was inhibited. Cell mobility was determined by a wound closure assay. Cells were placed onto 6-well plates at 80% confluence and cultured in serum-depleted media for 40 h. A wound was made by scraping the monolayer cells with a plastic pipette tip and fresh serum-free medium was replenished. Images of wound closure were photographed at 0, 16, 24 and 40 h post-scraping.

### Flowcytometry

Cells were trypsinized, resuspend in fresh medium followed by flowcytometry analysis. For cell cycle assay, cells were fixed with 70% ethanol and incubated on ice for 30 min. The cells were then suspended in PBS and treated with RNase A (final concentration 100ug/ml) at 37°C for 30 min. After removing RNase A, the cells were stained with propidium iodide (PI) at 5ug/ml for 10 min and the cell cycle was determined by flowcytometry analysis. For apoptosis assay, FITC Annexin V Apoptosis Detection Kit was used for staining the cells following product’s manual (BD bioscience). All flowcytometry data were analyzed using Summit v4.3 software.

### Immunohistochemical (IHC) staining

All the formalin-fixed paraffin-embedded (FFPE) slides were prepared and stained by the Pathology Core Facility at COH using a standard protocol. Antibodies used in this study were: rabbit polyclonal antibodyWNT5B (SAB2900204) (1:20 dilution, Sigma-Aldrich), mouse monoclonal antibody Myc (SC-40) (1:75 dilution, Santa Cruz Biotechnology) and rabbit monoclonal antibody MCL1 (ab32087) (1: 100 dilution, Abcam). All antibodies were titrated with negative and positive controls to obtain optimal staining.

### Electon microscope (EM)

The cells infected with shWNT5B or shCtl were collected in 3 days. The electron microscope was done in the core facility at COH following their standard protocol. It has been described in detail elsewhere [28]. The stained sections were subjected to Electron microscopy, which was done on an FEI Tecnai 12 transmission electron microscope equipped with a Gatan Ultrascan 2 K CCD camera.

### Oxygen consumption rate (OCR) and ATP measurement

The XF24 flux analyzer (Seahorse Bioscience) was used to measure OCR in 24-well microplates. Six thousand cells transduced with shCtl and 12000 cells infected with shWNT5B lenti-virus were seeded onto 24-well plates and incubated 3 days. The measurement, recording procedure and data analysis were described previously [29]. For cellular ATP measurement, we used ENLITEN ATP Assay System Bioluminescence Detection Kit (Promega Madison, WI). Briefly, the adherent cells in 6-well plate were collected by 2 mM EDTA in PBS on ice, TCA was add at final concentration of 1% and vortex vigorously for 10 sec. It was further diluted to 0.1% TCA by Tris-Acetate (pH = 7.75). The standard as well as the samples were serially diluted by dilution buffer (0.1% TCA/0.08 × PBS/0.9 × Tris-Acetate) and subjected to luminescence measurement.

### ELISA assay

To measure soluble WNT5B in patients’ serum, we used WNT5B ELISA Kit (USCN Life Science Inc.). The manufacture’s protocol was totally followed for preparing samples and all the reactions. The plate was read by SpectramaxPlus (Molecular Devices).

### Luciferase assay

ShWNT5B- or shCtl- virus transduced MDA-MB-231 cells were distributed into 12-well plates the day before transfection. Cells at 80% confluence were co-transfected with TCF-driven Topflash reporter plasmid (Millipore) (1 μg) and control Renilla luciferease (20 ng) using 2.5 μl of Lipofectamine 2000 (Invitrogen). Cells were lysed in 1X passive lysis buffer in 48 h and the supernatant was collected for Dual-luciferase activity measurement (Promega, Madison, WI). For each sample, firefly luciferase activity was normalized with an internal control, Renilla luciferase activity.

## Results

### WNT5B was upregulated in triple negative breast cancer

**Figure 1:**
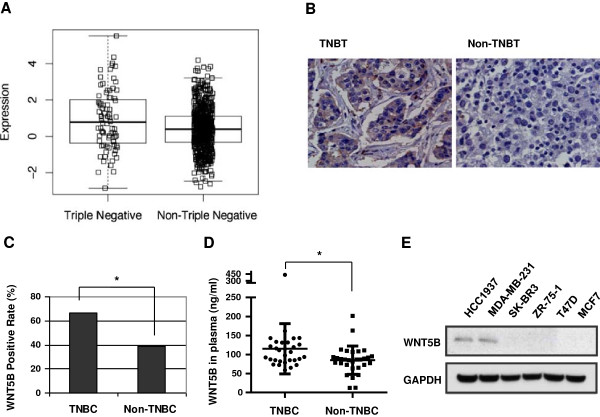
**WNT5B was upregulated in TNBC. (A)** Differential expression of WNT5B in TNBC and non-TNBC tissues was analyzed using cohorts of TCGA (n = 779), *P* = 0.0115. **(B)** Immunohistochemistry (IHC) staining of WNT5B in 21 TNBC and 121 non-TNBC tissues. **(C)** Statistical analysis of IHC staining of WNT5B in TNBC and non-TNBC tissues, positive staining rate was plotted, *P* = 0.0379. **(D)** ELISA analysis of WNT5B in 30 TNBC and 30 non-TNBC sera. Each dot represented the mean values 3 independent experiments. *P* = 0.0491. **(E)** Western blot of WNT5B in breast cancer cell lines. *P < 0.05, which was considered statistically significant.

We have previously performed microarray on 19 breast tumors, including 4 TNBC and non-TNBC tumors. We have reported the significant activation of WNT signaling in TNBC. To look for the ligand that might be important for TNBC tumorigenesis, we performed large scale public microarray data analysis instead of using our limited samples to achieve meaningful significance. As summarized in Additional file 1: Table S2; Title: Cohorts used in this study, cDNA microarry or RNA sequence data from 5 cohorts were collected [24-27] and used for comprehensive analysis of differential gene expression, metastasis and disease-free survival. The three datasets with appropriate metadata were analyzed to determine the differential expression between TNBC and Non-TNBC (Additional file 1: Table S2; Title: Cohorts used in this study). WNT5B mRNA was identified as one of the overexpressed gene in TNBC among 779 breast cancer tissues in TCGA data analysis (Figure 1a, Additional file 1: Table S1). The similar finding was observed in the analysis of other two analyses, which included 130 and 354 breast cancer tumors respectively (Additional file 1: Table S2; Title: Cohorts used in this study and Additional file 1: Figure S1). We validated the microarray results by immunohistochemistry (IHC) staining of WNT5B in breast cancer tissue array samples (Figure 1b). WNT5B was detected in 14 of 21 TNBC, while only 48 of 121 Non-TNBC tissues expressed WNT5B. Statistic analysis indicated that there was significant difference between TNBC and Non-TNBC (Figure 1c). Through autocrine or paracrine, WNT5B is secreted into the serum to function by binding to the cell surface receptor and co-receptor. Therefore, we randomly picked up 30 TNBC Versus 30 Non-TNBC stage IV patients and measured the soluble WNT5B level in their plasma. The average WNT5B in patients’ plasma was 115.01 ng/ml in TNBC; and 84.86 ng/ml in Non-TNBC. With approximately 30 ng/ml greater in TNBC than in Non-TNBC, and is a statically significant difference (Figure 1d). We further screened the WNT5B expression in breast cancer cell lines. RT-PCR results revealed that WNT5B predominantly expressed in TNBC-derived cell lines, HCC1937, MDA-MB-231 and BT-20; but not other Non-TNBC cell lines (Additional file 1: Figure S2) and this was confirmed with immunoblot analysis (Figure 1e). This finding suggested that WNT5B may play a role in TNBC.

### ShWNT5B led to impairment of cancerous features in TNBC cells

**Figure 2:**
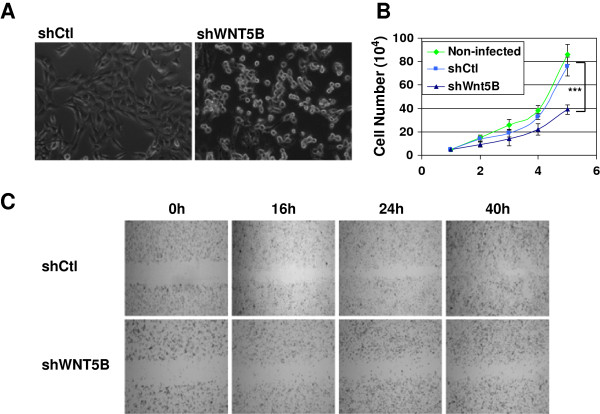
**Knockdown of WNT5B led to decreased functional change in MDA-MB-231 cells. (A)** Morphology alteration 3 days after shWNT5B was expressed. **(B)** Cell growth curve with WNT5B knockdown compared to control cells. Each point represents cell number as the mean ± SD (n = 3). Cells were observed for 5 days. ***P < 0.001 **(C)** ShWNT5B inhibited wound healing in MDA-MB-231 cells. Wound healing status at 0, 16, 24 and 40 h after wounding was photographed in MDA-MB-231 cells transduced with shWNT5B or shCtl. ShWNT5B attenuated mammary sphere formation in MDA-MB-231 cells. A representative mammary sphere image was taken (Left panel), and the sphere size greater than 50 um was enumerated. Scale bar: 20 um; the graph represents an average of 3 independent experiments. *P* = 0.0094, **P < 0.01 (Right panel).

To investigate the role of WNT5B plays in TNBC, we knockdown WNT5B by short hairpin RNA (shWNT5B) in TNBC-derived cell line MDA-MB-231 cells (MDA-MB-231/shWNT5B). The short hairpin RNA targeting non-mammalian sequence was served as control (shCtl). After 3 days expression of shWNT5B, MDA-MB-231 cell altered its morphology from spindle to round shape with poor attachment (Figure 2a). Flowcytometry was performed to determine the cell size. Decreased cell size was observed in MDA-MB-231/shWNT5B cells (Additional file 1: Figure S3A). We also measured the cell growth in shWNT5B and shCtl-infected MDA-MB-231 cells. It significantly decelerated in MDA-MB-231/shWNT5B cells as compared to shCtl-transduced cells or non-infected MDA-MB-231 cells (Figure 2b). The cell mobility was then examined by a wound healing assay. MDA-MB-231 cells infected with shCtl moved to the wound area within 16 h and completely closed the wound within 40 h; whereas in MDA-MB-231/WNT5B cells, the wound remained open, even after 40 h (Figure 2c). In proliferation assay, the cells transduced with shWNT5B demonstrated decreased proliferation comparing to control cells (Additional file 1: Figure S3B). These results indicate that WNT5B is a key factor to control cancer cell biology, especially in cell growth, motility, and tumorigenicity.

### ShWNT5B induced cell cycle arrest and caspase-independent cell death

**Figure 3:**
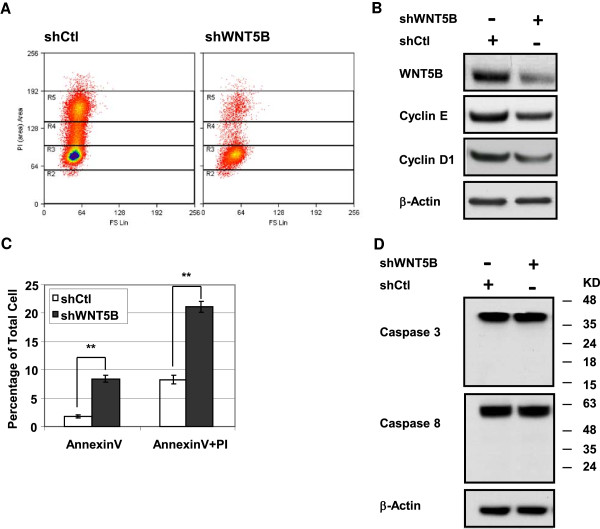
**ShWNT5B induced cell cycle arrest and caspase-independent apoptosis in MDA-MB-231 cells. (A)** Cells arrested in G0/G1 phase. Cells were infected with shCtl or shWNT5B, and the cell cycle was examined 3 days post-infection by flow cytometry after PI staining. **(B)** Immunoblot to confirm the WNT5B knockdown and to determine the expression of cyclin E and Cyclin D1. **(C)** ShWNT5B induced cell apoptosis. Cells were stained with Annexin V and PI followed by flow cytometry analysis. Bars represent the average of Annexin V or Annexin V plus PI cell number (n = 3). For Annexin V staining, *P* = 0.0032; for Annexin V plus PI staining, *P =* 0.0051. ** *P* < 0.01. **(D)** Immunoblot analysis of caspase-3 and caspase-8 based on the marker size to determine full length and cleaved proteins.

Given the cells growth worsened dramatically after WNT5B was inhibited, we assessed whether cell cycle transition was blocked. As it was shown in Figure 3a, cells with WNT5B knockdown underwent greatly increased G0/G1 cell cycle arrest. Cyclin E is an essential protein for the G1- to- S phase transition and it is regulated by Cyclin D1. To evaluate whether G0/G1 cell cycle arrest is due to the deregulation of Cyclin E and Cyclin D1, immunoblot was performed to examine Cyclin E and Cyclin D1 expression. As a result, with the suppression of WNT5B, enhanced reduction of Cyclin E and Cyclin D1 was detected (Figure 3b). On the other hand, with the inhibition of WNT5B, the cell survival length seemed to be shortened. We sought to determine whether it is caused by cellular apoptosis. The AnnexinV staining was conducted followed by flowcytometry analysis. The AnnexinV positive cell was 1.79% in shCtl-infected MDA-MB-231 cells, whereas it increased to 8.43% in the cells with WNT5B inhibition. The total of AnnexinV and PI positive cell was 8.30% in control cells and it went up to 21.11% in MDA-MB-231/shWNT5B cells. Both populations of AnnexinV positive cells and of AnnexinV plus PI positive cells were significantly increased with shWNT5B expression (Figure 3c). To identify whether the apoptosis induced by WNT5B knockdown is caspase-dependent, we did immunoblot analysis to determine the cleavage of Caspase 3/Caspase 8 in MDA-MB-231 cells. Neither the cleavage of Caspase 3 nor that of Caspase 8 was detected in MDA-MB-231/shWNT5B cells (Figure 3d). It clearly suggested that WNT5B depletion lead to a caspase-independent apoptosis, which is a feature of mitochondrial dysfunction. Moreover, the cell cycle analysis supported the impaired mitochondrial function as well, which was consistent with Dr. Finkel *et al*’s finding. In their experiments, they noticed a G0/G1 to S transition arrest through down-regulation of Cyclin E1 with the absence of ATP boost [30]. The observation of cell cycle alteration and caspase-independent apoptosis in MDA-MB-231/shWNT5B cells provided us a clue for characterization of mitochondria physiology.

### Knockdown of WNT5B attenuated mitochondrial biogenesis and oxidative phosphorylation (OXPHOS) in MDA-MB-231 cells

**Figure 4:**
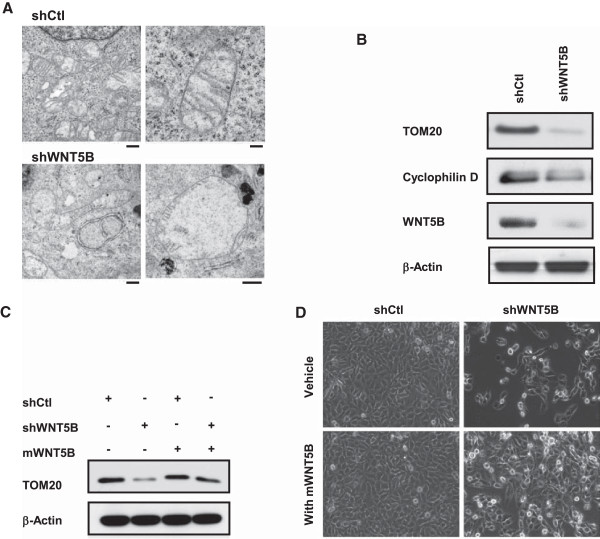
**WNT5B modulated mitochondrial homeostasis in MDA-MB-231 cells. (A)** Electron micrograph of cells expressing shWNT5B or shCtl. For left panel images, original magnification: x 4400, scale bars: 0.5 μm; for upper right image, original magnification: x 11000, scale bar: 0.2 μm; for lower right image, original magnification: x 6500, scale bar: 0.5 μm. It was stained with 2% uranyl acetate followed by Reynold’s lead citrate staining. **(B)** Immunoblot against TOM20, cyclophilin D and WNT5B. Diminished expression of TOM20 and cyclophilin D in shWNT5B transduced cells. **(C)** Western blot of TOM20 in MDA-MB-231/shWNT5B or control cells with or without the presence of 50 nM mWNT5B for 72 h. **(D)** Morphology change of MDA-MB-231/shWNT5B or control cells exposed to vehicle or 50 nM mWNT5B for 72 h.

**Figure 5:**
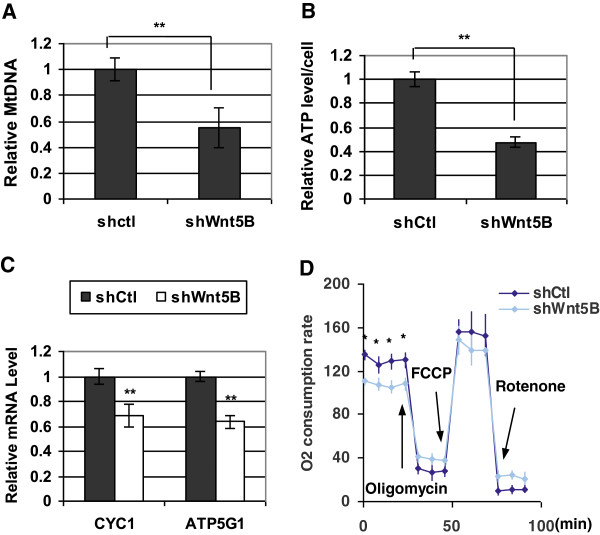
**shWNT5B suppressed mitochondrial biogenesis in MDA-MB-231 cells. (A)** qPCR analysis of mtDNA content. mtDNA was quantified by comparing the Cox2 gene to an intron of HBB in cells transduced with shWNT5B or shCtl virus. Bars represent the mean ± SD (n = 3), *P* = 0.0089. **(B)** Cellular ATP was extracted from shWNT5B- or shCtl-infected cells right before the assay, and the average single cell ATP level was calculated and graphed. The mean values represent 3 independent experiments. *P* = 0.0072. **(C)** RT-qPCR analysis of OXPHOS-related gene expression. CYC1 and ATP5G1 mRNA was detected and normalized with GAPDH. The results represent the mean of 3 independent experiments and 3 wells for each sample at each time point. **P = 0.0091 for CYC1 and *P* = 0.0084 for ATP5G1. **(D)** The OCR was measured and normalized by the cell number after oligomycin, FCCP and Rotenone were sequentially added to the cells. The values were plotted as the mean ± SD. *P* = 0.036. *P < 0.05; **P < 0.01.

The electron microscope was performed to study mitochondria. It was shown that mitochondrial number in MDA-MB-231/shWNT5B cells was much lower than that in shCtl-infected cells (Figure 4a Left panel, X4400). Moreover, the mitochondrial morphology was altered dramatically. Most mitochondria lost the typical internal tubular structure and severe swollen was frequent. They were no longer forming their original roundish rod shape; instead, multiple shapes were observed (Figure 4a). The mitochondrial size is much larger in shWNT5B expressing cells so that we had to decrease the magnification from X11000 to X6500 for viewing some large mitochondria in MDA-MB-231/shWNT5B cells. On the other hand, under the higher magnification, there were very little or no cristae observed in the mitochondria (Figure 4a Right panel) with WNT5B knockdown. The immunoblot was then carried out to verify the expression of proteins that are important for mitochondrial biology. As a result, the mitochondrial import receptor subunit TOM20 and the key regulator of mitochondrial permeability transition pore Cyclophilin D were barely detected with the inhibition of WNT5B (Figure 4b). We questioned whether worsened mitochondrial function could be prevented by WNT5B, we applied mouse recombinant WNT5B (mWNT5B) to MDA-MB-231/shWNT5B cells as well as control cells. The down-regulation of TOM20 in shWNT5B-transduced cells was avoided by mWNT5B (Figure 4c). In the meantime, the notable improvement of cell viability and growth were observed in mWNT5B-treated MDA-MB-231/shWNT5B cells (Figure 4d). These results highlighted the critical role that WNT5B played in mitochondrial physiology and implied that sufficient WNT5B was required for cell survival in MDA-MB-231 cells. We speculated that shWNT5B-triggered attenuation of cell viability and growth might be caused by compromised mitochondrial function in each cell. The mitochondrial dysfunction for an individual cell might be resulted from the reduction of mitochondrial number or dysfunction of each mitochondrion inside the cells; we conducted experiments to distinguish the conditions. We examined MtDNA by qPCR in MDA-MB-231/shWNT5B and control cells to evaluate the mitochondrial biogenesis first. Quantitative analysis uncovered that MDA-MB-231/shWNT5B cells showed a nearly twofold reduction in mitochondrial biogenesis compared to control cells (Figure 5a). Most of the cellular ATP is produced in the mitochondria; we detected the ATP level in MDA-MB-231 cells with or without WNT5B. The ATP generated by MDA-MB-231/shWNT5B cells was markedly dropped relative to control cells (Figure 5b). Since ATP was produced through oxidative phosphorylation, we further evaluated the expression of key mitochondrial OXPHOS genes, such as Cytochrome c 1 (CYC1) and ATP synthase γ subunit (ATP5G1). Consistent with the ATP level, the notable reduction of OXPHOS genes was observed in MDA-MB-231/shWNT5B cells (Figure 5c). Given that mitochondrial respiration is tightly coupled to the synthesis of ATP under normal biological conditions, we examined whether cellular oxygen consumption rate (OCR) altered as well. Significant reduction of basal OCR was seen in MDA-MB-231/shWNT5B cells compared to the control cells. However, there seemed to be no significant difference of reserve capacities. Interestingly, the offset difference after feeding oligomycin was very similar to that of adding rotenone, which suggested that there was no difference in proton leak. Rather, it was most likely due to the less response of mitochondria to the stimulations (Figure 5d). Given that the attenuation of mitochondrial biogenesis had been confirmed, it raised the possibility that the decreased mitochondrial mass rendered to compromised mitochondrial function in each cell. Collectively, the data implied that once WNT5B was down-regulated in MDA-MB-231 cells, the cells underwent cell cycle arrest and caspase-independent death caused by decreased mitochondrial mass. These data suggested that WNT5B was essential for mitochondrial physiology and thus critical for cell survival in TNBC.

### Possible mechanism for shWNT5B-induced suppresion of mitochondrial physiology

**Figure 6:**
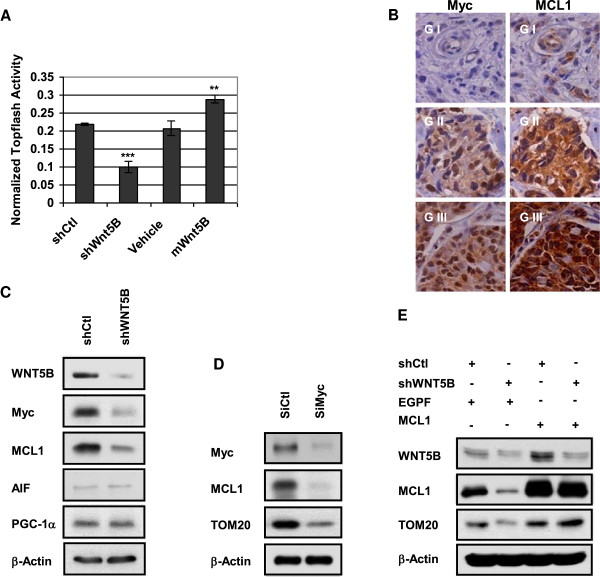
**Wnt5B initiated WNT/β-catenin to govern mitochondrial biogenesis through MCL1. (A)** Dual luciferase assay of TCF promoter activity. Twenty thousand cells were seeded into 12-well plates, and the cells were transduced with shCtl or shWNT5B lenti-particles and treated with vehicle control or 50 nM mWNT5B. The plasmid Topflash and Renilla luciferase were co-transfected into MDA-MB-231 cells at 24 h using lipofectamine 2000. The cells were harvested for the dual luciferase assay at 48 h. Bars represent the average luciferase activities normalized to internal control (n = 3). **P < 0.01; ***p < 0.001. **(B)** IHC staining of Myc and MCL1 in tissue array samples. FFPE tissue array slides were stained with Myc or MCL1 antibody, and the staining was graded into three different levels. Nuclear staining of Myc and cytosolic staining of MCL1 were observed. **(C)** Western blot of proteins related to mitochondrial structure and function. MDA-MB-231/shWNT5B and control cells were collected for cell lysates. The protein expression of MCL-1, AIF and PGC-1α was evaluated. **(D)** Western blot of Myc-regulated genes. MCL1 protein was inhibited with Myc knockdown by Myc siRNA; mitochondrial structural protein TOM20 also decreased. **(E)** MCL1 induced proteins in MDA-MB-231/shWNT5B cells. TOM20 was upregulated by MCL1 independent of WNT5B expression status.

To answer if WNT5B-mediated mitochondrial biogenesis controlled by WNT/β-catenin pathway, we carried out TCF promoter activity by dual luciferase assay. The result indicated that the promoter activity of TCF declined more than 50% in WNT5B inhibited cells relative to shCtl cells; while it enhanced approximately 30% in mWNT5B-treated MDA-MB-231 cells compared to cells treated with vehicle control (Figure 6a). Once WNT/β-catenin pathway was identified as a pathway that was triggered by WNT5B, we performed correlation study of WNT5B-related WNT/β-catenin pathway target genes in 884 breast tumor samples; Myc was demonstrated a significant correlation with WNT5B (Additional file 1: Figure S4a). We further conducted genome wide survey of WNT5B-related genes in the same sample set and MCL1 was listed as the candidate that is positively correlative with WNT5B expression (Additional file 1: Figure S4b). Since MCL1 was an anti-apoptotic protein, which was lately identified as the key regulator of mitochondrial function [31]. Therefore, we hypothesized that WNT5B might govern mitochondrial biogenesis via MCL1 that was modulated by WNT/β-catenin target gene, Myc. In order to determine the correlation of Myc with MCL1, IHC staining of Myc and MCL1 was performed in 142 breast tumor tissue array samples and the staining was graded as weak positive (G I), medium positive (G II) and strong positive (G III). The correlative analysis of the staining revealed that the staining grade of the two proteins was consistent in 98 out of 142 tumor tissues, which represented a significant correlation (Figure 6b, Additional file 1: Table S3). These clinical data provided strong evidence that WNT5B might modulate mitochondrial physiology through MCL1, which was mediated by WNT/β-catenin pathway target gene, Myc. To further confirm this hypothesis, we conducted immunoblot and the results showed that shWNT5B remarkably reduced the expression of Myc and MCL1 in MDA-MB-231/shWNT5B cells relative to control cells. We also assessed if WNT5B controlled mitochondrial biogenesis through the other proteins known to contribute to mitochondrial biogenesis, such as PGC-1a and AIF. As a result, there is no expressional change of these two proteins between MDA-MB-231/shWNT5B and control cells (Figure 6c). We next verified whether Myc regulated the expression of MCL1 in MDA-MB-231 cells. We diminished the expression of Myc by SiRNA targeting Myc. As illustrated in Figure 6d, MCL1 level attenuated with the suppression of Myc. This was in accordance with recent report, in which Myc was recognized as a gene that could direct transcription of MCL1 [32], Moreover, inhibition of Myc decreased the expression of mitochondrial structural protein, TOM20 as well (Figure 6d). Finally, we overexpressed MCL1 in MDA-MB-231/shWNT5B cells to evaluate if the impaired TOM20 expression could be prevented by MCL1. As a result, the suppressed TOM20 was brought to the level of control cells after MCL1 was forcedly overexpressed (Figure 6e). Taken together, the data implied that WNT5B triggered WNT/β-catenin signaling to maintain mitochondrial mass and function through Myc-induced MCL1 expression.

### Clinical significance of WNT5B in metastasis and disease-free survival of TNBC

**Figure 7:**
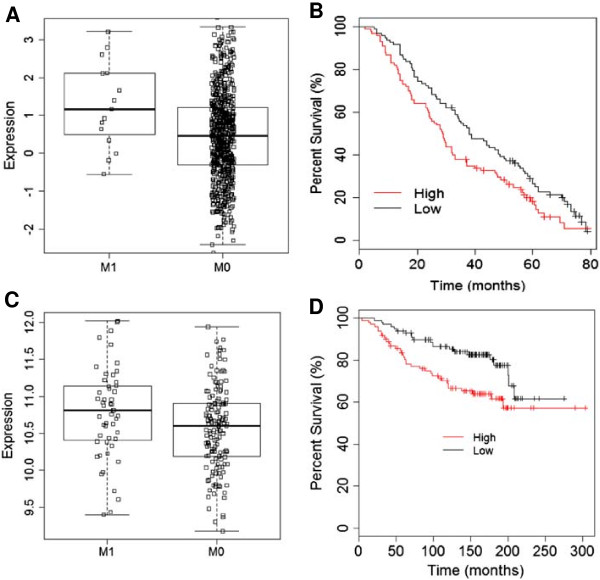
**Clinical correlation of WNT5B with metastasis and disease-free survival. (A)** Differential expression of WNT5B in metastasis (M1) and non-metastasis (M0) groups using TCGA microarray data. *P* = 0.0221 **(B)** Disease-free survival analysis in the high WNT5B and low WNT5B groups using the combined data pulled out from the studies by Desmedt et al. and Wang et al. Probe, WNT5B_221029_s_at, n = 198, *P* = 0.0212. **(C)** Differential expression of MCL1 in metastasis (M1) and non-metastatsis (M0) groups using microarray data of Desmedt et al. **(D)** Disease-free survival analysis in the high MCL1 and low MCL1 groups using same microarray data collected from the study of Desmedt et al.

WNT5B was upregulated in TNBC and TNBC derived cell lines. Experimental data demonstrated its critical role in TNBC cell, MDA-MB-231. We then asked the clinical significance of WNT5B in TNBC patients. Again, we conducted large scale analysis using public domain microarray data (Additional file 1: Table S2) to evaluate if WNT5B expression was associated with metastasis and survival. Because of the small sample size of TNBC in each cohort and the limited availability of certain metadata for comparing metastatic vs. non-metastatic expression or disease-free survival within TNBC patients. Therefore, we extended the analysis to the entire breast cancer population in the studies that the detailed metastasis and survival information was available. Interestingly, in both cohorts of expO and TCGA, the metastatic patients showed significantly higher expression of WNT5B (Figure 7a and Additional file 1: Figure S5). With this *in vivo* data strongly supported our *in vitro* findings; we sought to study whether WNT5B is ultimately associated with survival. The data demonstrated that the group with abundant WNT5B was related to lower disease-free survival rate compared to patients with lower WNT5B level in each study. The combination of the two cohorts achieved even better significance in the correlation of WNT5B with disease-free survival (Figure 7b Additional file 1: Figure S6). Similar analysis of MCL1 in the study of Desmedt et al. yielded better significance. It might be because of the higher specificity of MCL1 by comparing with its upstream gene, WNT5B. Collectively, both the *in vitro* and *in vivo* results suggested that WNT5B-initiated MCL1 signaling dominantly controlled the overall outcome of breast cancer patients, especially in TNBC.

## Discussion

We have previously reported that aberrant activation of WNT signaling contributed to proliferation and metastasis in TNBC cells and in animal model [11]. To carefully address the wider role of WNT signaling playing in the TNBC, we extended the study population from the data in our institute to the public arrays. Firstly, WNT5B was identified as the possible ligand for activation of WNT signaling in TNBC. In the functional study, we found that WNT5B played a crucial role for TNBC cells. It empowered cell growth through regulation of the cellular energy plant, mitochondria. Most importantly, this delicate regulation by WNT5B didn’t limited in a particular cell model; it was fundamentally associated with patients’ metastasis and disease-free survival in a larger population with breast cancer. These strong evidences highlighted the promising effect that WNT5B exerts on TNBC.

The WNT effect is highly dynamic and tissue context-specific in human cancers. For instance, the elevated WNT3A promotes the growth of myeloma cells in vitro [33] and prostate tumor in mouse model [34], while it dramatically decreases the growth of melanoma cells transplanted in the mice [35]. Most likely, each WNT exhibits unique sensitivities and the response upon a particular tissue-derived cancer, which might be true for WNT5B in TNBC. Recently, it has been noted that WNT signal promoted mitochondrial biogenesis in mouse skeletal myoblasts [36]; it was also observed that mitochondrial function and oxidative phosphorylation were impaired in hepatocytes of β-catenin knockout mice [37]; and the adipocyte mitochondrial metabolism was suppressed through WNT inhibition [19]. Collectively, we speculate that convergence on the mitochondria might be a mechanism of WNT controlling diverse process in some cancer cells. Despite the multitude of reports, the mechanism of how WNT modulate mitochondrial physiology in TNBC remains unexplored. In the current study, MCL1 was verified as the responsive protein which opposed cell death through controlling mitochondrial homeostasis. Among the Bcl-2 pro-survival protein family members, MCL1 was the one that raised particular attention because of its high expression in extensive cancer subtypes and its functions that extended beyond apoptosis regulation, but contributed to diverse biological process, such as malignancy and autophagy [38,39]. Increased MCL1 levels in cancer cells can result from elevated transcription or translation and decelerated degradation [40,41]. A genome-wide study of somatic copy number amplification (SCNA) uncovered that MCL1 was enriched in over 3000 tumor samples collected from 26 histological types. The increased copy number of MCL1 was found in more than 10% of cancers, but the amplification was higher in lung and breast cancers [42]. Recent research progression of TNBC indicated that Myc and MCL1 are both upregulated in TNBC and they play important role in cell survival [43]. In the current study, we demonstrated that WNT5B-stimulated WNT/β-catenin signaling held MCL1 at high level via its target protein, Myc. It was also reported that GSK3 controlled MCL1 degradation by phosphorylation of MCL1 for ubiquitylation-dependent degradation. Impaired phosphorylation of GSKs induced by activation of WNT/β-catenin might corporate with Myc to stabilize MCL1 in TNBC. We will address it in the future. Taken together, our study provided wider insight into the deeper role of WNT5B-triggered WNT/β-catenin signaling; it might regulate breast tumor progression and outcome by modulating mitochondrial physiology through MCL1.

## Conclusions

Taken together, the data suggest that WNT5B plays an important role in aberrant activation of WNT canonical pathway in TNBC. Inhibition of WNT5B induces cell cycle arrest and caspase-independent apoptosis, which is caused by attenuated mitochondrial biogenesis. WNT5B modulates mitochondrial biogenesis through MCL1, which is regulated by WNT/β-catenin responsive gene, Myc. These findings provide promising evidences to target WNT5B-indeced WNT/β-catenin signaling in TNBC.

## Abbreviations

TNBC: Triple negative breast cancer; MtDNA: Mitochondrial DNA; OXPHOS: Oxidative phosphorylation; HER2: Epidermal growth factor receptor 2; IHC: Immunohistochemistry; mWNT5B: Mouse recombinant WNT5B; CYC1: Cytochrome c 1; ATP5G1: ATP synthase γ subunit; OCR: Cellular oxygen consumption rate; TOM20: Translocase of outer mitochondrial membrane 20 homolog (yeast).

## Competing interests

The authors declare that they have no competing interests.

## Authors’ contributions

LY, DA, and YY designed the research; LY, AAP, SF, JL, YW, BY, YRC, XL, HZ, SZ, performed the research; LY, CW, YRC, ZL, DA, and YY analyzed the data; and LY, and CW, YRC, and YY wrote the paper. All authors read and approved the final manuscript.

## Pre-publication history

The pre-publication history for this paper can be accessed here:

http://www.biomedcentral.com/1471-2407/14/124/prepub

## Supplementary Material

Additional file 1: Figure S1 WNT5B is upregulated in TNBC from microarray analysis. **Figure S2.** WNT5B was upregulated in TNBC. RT-PCR of WNT5B in breast cancer cell lines. **Figure S3.** Knockdown of WNT5B led to cell size change and decreased cell proliferation in MDA-MB-231 cells. (A) Cells were transduced with shWNT5B lentiviral particles, and cell size change was measured by flow cytometry 3 days after shWNT5B expression. (B) 2000 cells were seeded into 96-well plate and infected with shCtl or shWNT5B virus. The cell proliferation were evaluated in 3 days after infection. **P < 0.01. **Figure S4.** Statistical analysis of WNT5B with its correlated genes. (A) WNT5B expression was significantly correlated with Myc, *P* = 3.7e-6, r = 0.15. (B) WNT5B level was statistically correlated with MCL1, *P* = 5.8e-9, r = 0.19. The data were collected from the public microarray TCGA in which 779 breast tumors were studied in the cohort. **Figure S5.** Clinical correlation of WNT5B with metastasis. **Figure S6.** Clinical correlation of WNT5B with disease-free survival. (A) Disease-free survival analysis in the high WNT5B and low WNT5B groups using the data pulled from the studies by Desmedt et al. n = 127, *P* = 0.0234. (B) Same analysis using data pulled from Wang et al. n = 71, *P* = 0.0311. Both studies used probe WNT5B_221029_s_at. **Table S1.** Primers used in this study. **Table S2.** Cohorts used in this study. **Table S3.** IHC staining of Myc and MCL1. Click here for file
