# Supplementary material for: Wnt modulates MCL1 to control cell survival in triple negative breast cancer
Source: BMC Cancer. 2014 Feb 24;14:124. doi: 10.1186/1471-2407-14-124 (PMC3936852; doi:10.1186/1471-2407-14-124)
Supplement: Additional file 1: Figure S1 — WNT5B is upregulated in TNBC from microarray analysis. Figure S2. WNT5B was upregulated in TNBC. RT-PCR of WNT5B in breast cancer cell lines. Figure S3. Knockdown of WNT5B led to cell size change and decreased cell proliferation in MDA-MB-231 cells. (A) Cells were transduced with shWNT5B lentiviral particles, and cell size change was measured by flow cytometry 3 days after shWNT5B expression. (B) 2000 cells were seeded into 96-well plate and infected with shCtl or shWNT5B virus. The cell proliferation were evaluated in 3 days after infection. **P < 0.01. Figure S4. Statistical analysis of WNT5B with its correlated genes. (A) WNT5B expression was significantly correlated with Myc, P = 3.7e-6, r = 0.15. (B) WNT5B level was statistically correlated with MCL1, P = 5.8e-9, r = 0.19. The data were collected from the public microarray TCGA in which 779 breast tumors were studied in the cohort. Figure S5. Clinical correlation of WNT5B with metastasis. Figure S6. Clinical correlation of WNT5B with disease-free survival. (A) Disease-free survival analysis in the high WNT5B and low WNT5B groups using the data pulled from the studies by Desmedt et al. n = 127, P = 0.0234. (B) Same analysis using data pulled from Wang et al. n = 71, P = 0.0311. Both studies used probe WNT5B_221029_s_at. Table S1. Primers used in this study. Table S2. Cohorts used in this study. Table S3. IHC staining of Myc and MCL1. [file 1471-2407-14-124-S1.pdf]

Supplemental Data

Supplementary Figure 1

Chin **ExpO**

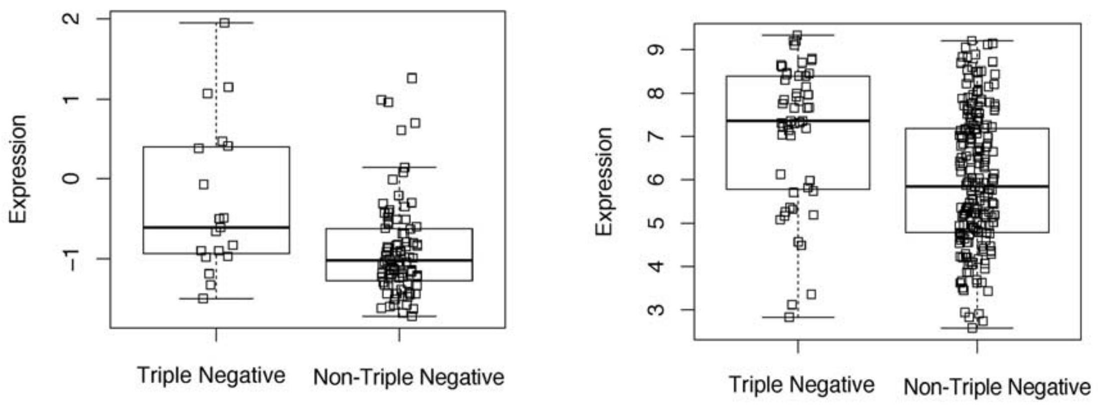

Supplementary Figure 1. WNT5B is upregulated in TNBC from microarray analysis. Differential expression of WNT5B in triple negative and non-triple negative patients was analyzed. Both studies used the probe WNT5B_221029_s_at and *P* value was determined by *t* - test in R. *P = 0.0155* in Chin cohort (n = 130) and *P* = 5.09e-05 in expO study (n = 354).

Supplementary Figure 2

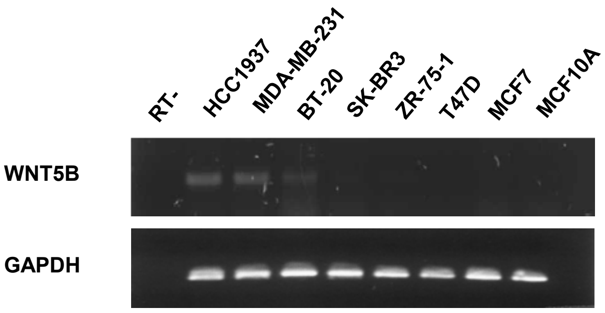

Supplementary Figure 2. WNT5B was upregulated in TNBC. RT-PCR of WNT5B in breast cancer cell lines. HCC1937, MDA-MB-231 and BT20 were TNBC-derived cell lines. The others were non-TNBC-derived cell lines.

Supplementary Figure 3

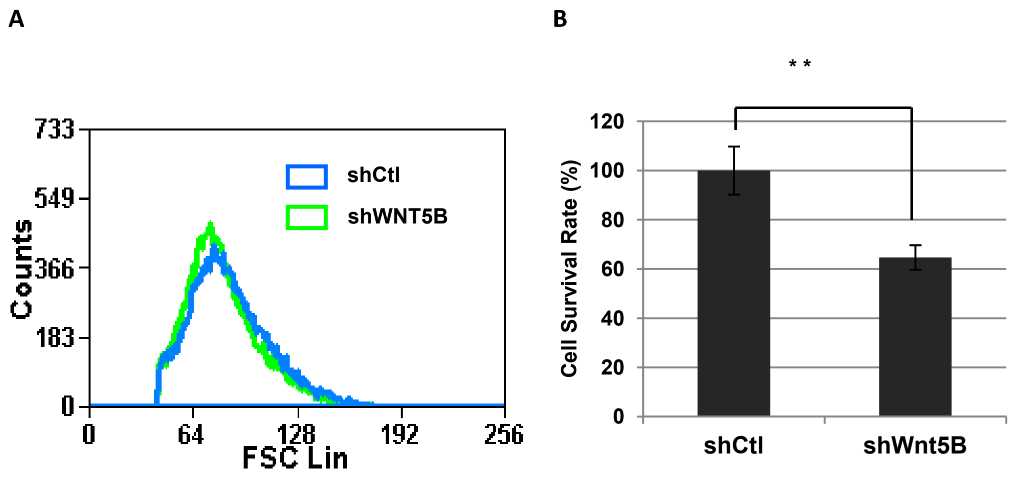

Supplementary Figure 3. Knockdown of WNT5B led to cell size change and decreased cell proliferation in MDA-MB-231 cells. (A) Cells were transduced with shWNT5B lentiviral particles, and cell size change was measured by flow cytometry 3 days after shWNT5B expression. (B) 2000 cells were seeded into 96-well plate and infected with shCtl or shWNT5B virus. The cell proliferation were evaluated in 3 days after infection. ** $P < 0.01$.

Supplementary Figure 4

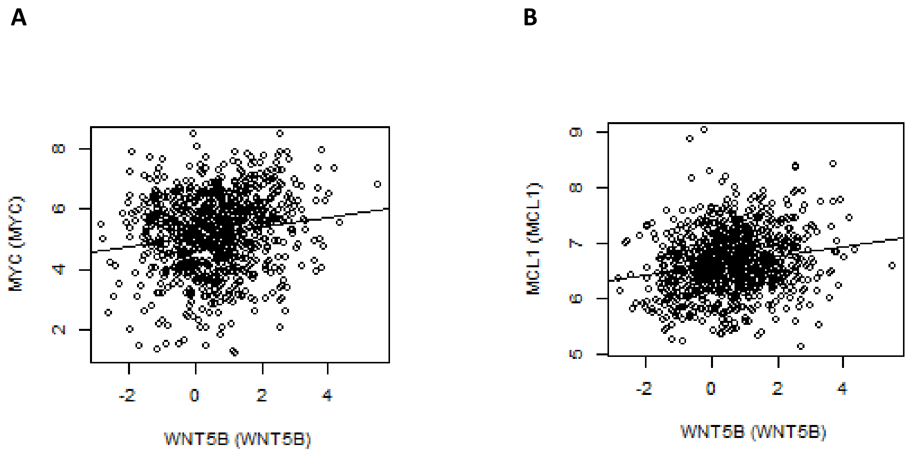

Supplementary Figure 4. Statistical analysis of WNT5B with its correlated genes. (A) WNT5B expression was significantly correlated with Myc, P = 3.7e-6, r = 0.15. (B) WNT5B level was statistically correlated with MCL1, P = 5.8e-9, r = 0.19. The data were collected from the public microarray TCGA in which 779 breast tumors were studied in the cohort.

Supplementary Figure 5

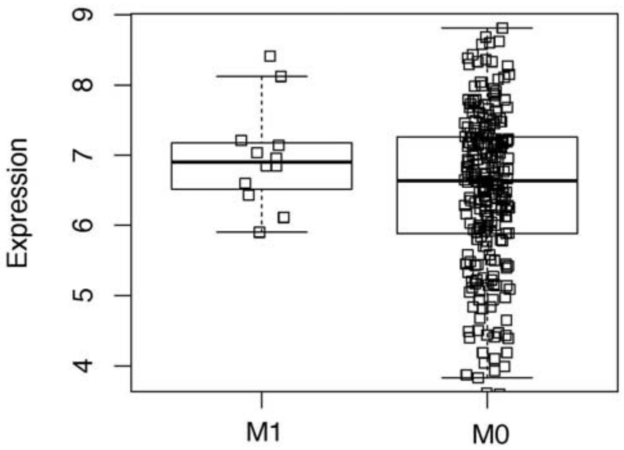

Supplementary Figure 5. Clinical correlation of WNT5B with metastasis. Differential expression of WNT5B in metastasis (M1) and non-metastasis (M0) groups for the samples in expO study. *P* = 0.0432; probe, WNT5B_223537_s_at.

Supplementary Figure 6

A

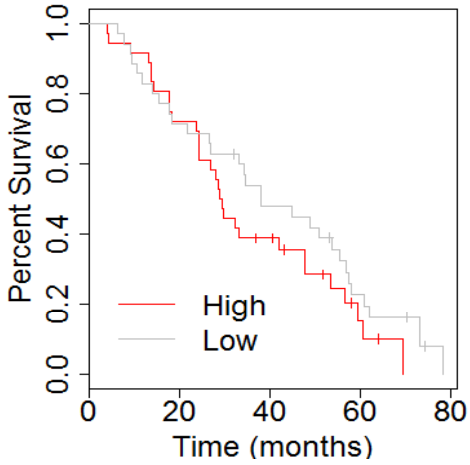

B

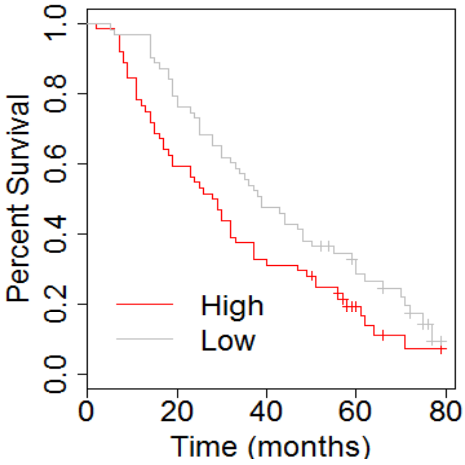

Supplementary Figure 6. Clinical correlation of WNT5B with disease-free survival. (A) Disease-free survival analysis in the high WNT5B and low WNT5B groups using the data pulled from the studies by Desmedt et al. n = 127, *P* = 0.0234. (B) Same analysis using data pulled from Wang et al. n = 71, *P* = 0.0311. Both studies used probe WNT5B_221029_s_at.

Supplementary Table1. Primers used in this study

Primer	Gene Description	Sequence(5' → 3')
ATP5G1 R	ATP synthase subunit b'	GCCAAGAATGGCATAGGAGA
ATP5G1 F	ATP synthase subunit b'	ACATTGACACAGCAGCCAAG
CYC1 R	cytochrome c-1	TATGCCAGCTTCCGACTCTT
CYC1 F	cytochrome c-1	CCAGCTACCATGTCCCAGAT
MtDNA COX2 R	cytochrome c oxidase subunit II	TAAAGGATGCGTAGGGATGG
MtDNA COX2 F	cytochrome c oxidase subunit II	TTCATGATCACGCCCTCATA
GAPDH-R	glyceraldehyde-3-phosphate dehydrogenase	CAGACCCTAGAATAAGACAGG
GAPDH-F	glyceraldehyde-3-phosphate dehydrogenase	ACTGCCAACGTGTCAGTGGTG
HBB int R	hemoglobin, beta	AATCCAGCCTTATCCCAACC
HBB int F	hemoglobin, beta	TATCATGCCTCTTTGCACCA
Wnt5B3eF	wingless-type MMTV integration site family, member 5B	AGACTGGCATCAAGGAATGC
Wnt5B4eR	wingless-type MMTV integration site family, member 5B	GCTGATGGCGTTGACCAC

Supplementary Table 2. Cohorts used in this study

Cohort name	Number of samples	Publication* (PMID)	Analysis		
			TNBC DEG *	Metastasis	Disease-free survival
Chin	130	17157792	X		
expO	354	GSE2109	X	X	
TCGA	779	23000897	X	X	
Desmedt	198	17545524			X
Wang	286	15721472			X

* GEO ID was used for expO (Expression project for Oncology); DEG: Differential expression gene

Supplementary Table 3. IHC staining of Myc and MCL1

Myc	MCL1: G I	MCL1: G II	MCL1: G III
G I	2	2	43
G II	4	41	14
G III	27	5	4

Correlation study of Myc and MCL1 for IHC staining.
Correlation study of Myc and MCL1 for IHC staining.
IHC staining was carried out in 142 breast tumor tissue array FFPE specimens. The staining was graded as G I, weakest staining; G II, moderate staining; and G III, strongest staining. The stained slides were reviewed, and the grade was given to each specimen for each staining. The correlation was analyzed by using correlation test function in R. P = 2.2e-16, r = 0.73.
